# Dialogical Family Guidance (dfg)—Development and implementation of an intervention for families with a child with neurodevelopmental disorders

**DOI:** 10.1002/nop2.627

**Published:** 2020-09-17

**Authors:** Diana Cavonius‐Rintahaka, Anna Liisa Aho, Eva Billstedt, Christopher Gillberg

**Affiliations:** ^1^ Gillberg Neuropsychiatry Centre Institute of Neuroscience and Physiology University of Gothenburg Sahlgrenska Academy Gothenburg Sweden; ^2^ Child Psychiatry Neuropsychiatric Unit Helsinki University Hospital Helsinki Finland; ^3^ Faculty of Social Sciences Nursing Science University of Tampere Tampere Finland

**Keywords:** dialogue, family, implementation, intervention, neurodevelopmental disorders, parents

## Abstract

**Aim:**

To describe the development and implementation of a Dialogical Family Guidance (DFG) intervention, aimed at families with a child with neurodevelopmental disorders (NDD).

**Design:**

The DFG components are presented and the content of a DFG training course. Professionals' experiences after the DFG training were evaluated.

**Methods:**

Dialogical Family Guidance development phases and implementation process are examined. The Revised Standards for Quality Improvement Reporting Excellence checklist (SQUIRE 2.0) was used to provide a framework for reporting new knowledge.

**Results:**

The DFG training course seemed to increase possibilities of a more independent role as a nurse to deliver the DFG family intervention. The project showed that the use of dialogue can be difficult for some professionals. Analysis of the questionnaire completed after DFG training reported a high level of satisfaction. DFG training offered a new approach to deliver knowledge and understanding to families using dialogue, including tailored psychoeducation and emotional and practical guidance.

## 1 INTRODUCTION

Articles presenting guidelines for implementation processes and detailed frameworks offer important sources to promote knowledge between professionals (Breimaier, Heckemann, Halfens, & Lohrmann, 2015; Hickey et al., 2016; Kwak, Wahlin, Stigmar, & Jensen, 2017). Systematic development and implementation of interventions is essential for their interpretation, and they need to be carefully planned and designed. The implementation of evidence‐based interventions is crucial to professional nursing, but more research is still needed. The professional responsibility of nurses is aimed at providing high‐quality nursing interventions and, in that way, positive health outcomes (van Achterberg, Schoonhoven, & Grol, 2008; Whittemore & Grey, 2002). However, it is important that the development and implementation of these interventions are evaluated. Thus, implementation research studies systematically document how an intervention has been carried out in clinical practice (Goldenhar, LaMontagne, Katz, Heaney, & Landsbergis, 2001). This article describes the development and implementation process of a family intervention called Dialogical Family Guidance (DFG) aimed at families with a child with NDD (neurodevelopmental disorders). The DFG‐educational elements are also presented.

Neurodevelopmental disorders is a general appellation to describe neurological and psychiatric disorders with an early onset in childhood. Neurodevelopmental disorders includes learning and language disorders, motor coordination disorders, intellectual disabilities, autism spectrum disorders (ASD), attention‐deficit/hyperactivity disorder (ADHD), tic disorders and oppositional defiant disorder (ODD). Common comorbidities are sleeping disorders, feeding problems and various sensory processing problems. A change in symptom/developmental profile may occur during the childhood period which is further emphasized in the concept of ESSENCE (Early Symptomatic Syndromes Eliciting Neurodevelopmental Clinical Examinations) created by professor Gillberg from University of Gothenburg. The ESSENCE concept covers NDD, and problems/symptoms not meeting the criteria for a certain NDD diagnosis (Gillberg, 2010; Thapar, Cooper, & Rutter, 2017). A family intervention model designed with a focus on the individual family's needs and questions has been advocated for this particular group (Cavonius‐Rintahaka, Aho, Voutilainen, Billstedt, & Gillberg, 2019).

### 1.1 Background

Parenting stress, family dynamics and family function surrounding the child´s disorder should be considered when developing interventions for families with a child with NDD (Ho, Chien, & Wang, 2011; Wiener, Biondic, Grimbos, & Herbert, 2016). Subsequently, there is also a need to translate the heightened stress, illness and psychiatric problems occurring in parents of children with NDD into effective interventions (Dykens, 2015). Dykens (2015) points out that both parents and the siblings in the family need accurate and targeted guidance and information. It is known, for example, that ADHD is associated with problematic family functioning, including greater stress in the family, higher rates of parental psychopathology and conflicted parent–child relationships, and this appears to exacerbate in children with comorbid oppositional and conduct disorders (Deault, 2010). Also, autism symptom severity is significantly correlated with maternal stress (Duarte, Bordin, Yazigi, & Mooney, 2005).

When providing support to parents with children with NDD, the focus needs to be on the entire family and not only on the child with the diagnosis. However, it is also noticed that there are differences between mother's and father's ways of coping with their child's diagnosis and stressful life events. Parents have different personalities and parenthood behaviours. Studies highlight the need to translate parents' heightened stress and siblings' needs, to give accurate and targeted guidance and offer effective interventions to strengthen the well‐being of the whole family (Duarte et al., 2005; Falk, Norris, & Quinn, 2014).

Open dialogue was originally developed as a method for healthcare teams to help adult psychosis patients in Finland but has since been implemented in different countries and modified to fit different healthcare organizational needs. Consequently, open dialogue no longer seems to be a therapeutic method but rather the ability to see the polyphonic nature of the client's reality. The base from which to offer professional help is realized by listening carefully to what the client and family members have to say (Anderson, 2002; Buus et al., 2017; Rober, 2010; Seikkula, Arnkil, & Eriksson, 2003). By using the dialogue approach and supporting dialogue in conversation, nurses and other professionals can help families with a child with NDD get through distressing life events and demands. Especially, giving grass‐root attention to the voices of individuals and families, speaking from experience is important over the treatment (Post, Pomeroy, Keirns, Cover, & Dorn, 2017). As a further consideration, NDD symptoms and diagnoses are strongly heritable, and subsequently, more than one family member can have special needs or special difficulties that fall under the NDD symptom umbrella (Lichtenstein, Carlstrom, Rastam, Gillberg, & Anckarsater, 2010; Thapar & Cooper, 2016).

There is no doubt that providing education increases knowledge and positive attitudes and behaviours towards individuals with NDD. To accomplish dialogue, professionals themselves need to adopt a positive and cooperative attitude. This attitude includes aspects such as understanding, empathy, flexibility, a high motivation to cooperate with families and a willingness to help them (Anderson, 2002; Buus et al., 2017; Seikkula et al., 2003). It is known, for example, that primary caregivers of adolescents with ADHD experience better quality of life, family functioning and parental coping after Therapeutic Conversation Intervention, and therefore, this intervention has been recommended for nurses in hospitals and at healthcare centres, where ADHD services are provided (Gisladottir & Svavarsdottir, 2017). Negative attitudes and a lack of time can be a threat to parents' confidence. Thus, the attitude of the parents and their willingness to cooperate is also an important factor when trying to achieve optimal results.

Cavonius‐Rintahaka et al. (2019) conducted a pilot study about families' health, functionality, hopes and expectations and confirmed that families with a child with NDD seemed not to get the help they expected from professionals. Notably, parents, both hoped and expected professionals to listen, have dialogue and give attention to the entire family. Therefore, the Dialogical Family Guidance intervention is an important step forward in trying to meet parental and family needs.

## 2 METHODS

The aim of this paper is to describe the development and the implementation process of the Dialogical Family Guidance (DFG) family intervention. Important components of the intervention and the implementation process into the clinical setting are presented, including the DFG‐educational process developed for professionals. A post‐training evaluation was carried out for professionals who had taken part in DFG training to collect data about their satisfaction concerning the training they had received. A tailored questionnaire with 10 questions about DFG training (Likert scale 1–7) was completed after the training by 26 professionals. One open‐ended question was included. The quantitative data were analysed by using the SPSS statistical programme, and the results of the open‐ended question are presented as a summary. The Revised Standards for Quality Improvement Reporting Excellence checklist (SQUIRE 2.0) has been used to provide framework for reporting new knowledge about how to improve health care (Ogrinc et al., 2015) and has also been employed in this study (Appendix S1).

### 2.1 Literature review

A review of the literature presents psychoeducation as a commonly used and valuable intervention for families with a child with NDD (Nussey, Pistrang, & Murphy, 2013). It has been defined as a systematic and didactic approach, adequate for informing patients, relatives, school staff, etc., about the condition and for implementing educational programmes related to a child's disorder. According to the literature, effective psychoeducation is carried out by a sensitive and sympathetic therapist, lasting approximately 60–90 min and including 4–6 sessions (Bauml, Frobose, Kraemer, Rentrop, & Pitschel‐Walz, 2006). Studies of psychoeducation show that children and adults with NDD, and families and teachers, benefit from this intervention regarding their psychosocial well‐being (Antai‐Otong & Zimmerman, 2016; Ferrin et al., 2014; Hirvikoski et al., 2017; Jackson, Liang, Frydenberg, Higgins, & Murphy, 2016; Nussey et al., 2013; Richardson et al., 2015; Sonuga‐Barke, Daley, Thompson, Laver‐Bradbury, & Weeks, 2001; Tonge, Brereton, Kiomall, Mackinnon, & Rinehart, 2014).

Although many psychosocial interventions have been tested as effective (Mazzucchelli, Jenkins, & Sofronoff, 2018; Potvin, Prelock, & Savard, 2018), the development processes and theories behind these interventions are not always described nor published. Also, the information about how the intervention has been implemented into clinical setting can be missing and the new intervention can be presented more as a project plan. Occasionally, there is too little said about the process of implementation design. The literature also offers us examples of psychoeducational interventions for families with a child with NDD (see Table 1 for examples). However, it seems that any dialogue or dialogical elements are often overlooked or missing because dialogue is only randomly mentioned. It should be noticed that although many interventions seem to offer a family intervention, they often only target the parents in the family and are thus parent‐mediated. This means that it is much easier to find “parenting programmes” than “family interventions” where siblings are in focus alongside the parents. However, earlier studies highlight similar important elements that are included in DFG, for example collaboration, discussion, family‐identified goals, family uniqueness and reflective listening. Given the inclusions and omissions seen in the literature, a family intervention that target all family members and which includes dialogue along with psychoeducation and gives the family tools to cope with their daily life is needed. To offer readers a perspective of the elements covered across previous literature, a summary of seven papers including examples of various intervention aspects is presented in Table 1.

**Table 1:** Summary of seven interventions aimed towards families with a child with NDD

Author	Intervention	Content
Potvin et al., 2018. USA	Coaching in context (CinC)	Family‐driven support for children with autism and their families combining coaching and context therapy. Professionals coach the whole family, and the intervention is said to be family‐driven. However, it is actually a parent‐mediated structured process. Parents deliver the intervention in practice to their child. This involves families in goal setting, designing, implementing and evaluating during the process. The coach gets support from an inter‐professional team, and this is called the “key” in this process. This is a descriptive paper, and CinC has not been tested.
Dunn, Cox, Foster, Mische‐Lawson & Tanquary,^2012^. USA	Occupational therapy contextual intervention	This ten‐session Occupational Therapy Contextual Intervention is aimed to improve participation in everyday life for children with autism spectrum disorders and develop parent competence. Combines context therapy with coaching elements and is provided by occupational therapists. Effectiveness was evaluated using pre‐test–post‐test design. Results indicated that parents felt more competent and children increased participation in everyday life. This intervention is mainly about coaching parents in daily life to achieve their own goals concerning their family.
Oruche, Robb, Aalsma, Pescosolido, Brown‐Podgorski & Draucker, 2017. USA	Multiple caregiver group	For caregivers of adolescents with disruptive behaviours. Six‐week caregiver group intervention for primary caregivers of adolescents diagnosed with oppositional defiant disorder or conduct disorder. Aim of this intervention is to increase primary caregivers' self‐efficacy in managing interactions within and outside the family. This is a descriptive paper.
Mazzucchelli et al., 2018. Australia	Building bridges triple P (BBTP)	Eight‐week long group format parenting programme for parents of adolescents with autism spectrum disorders. The aim is to study the feasibility of the BBPT initiative targeted at the needs of parents of adolescents with a developmental disability. Study results are, for example parents' decreased symptoms of depression and stress, and increased parenting confidence. Results provide preliminary support and acceptability for BBTP.
Gisladottir & Svavarsdottir, 2017. Iceland	Therapeutic conversation intervention (TCI)	Combination of group and individual sessions focusing on reinforcing, improving and sustaining an active family life for families with adolescents with ADHD, targeting caregivers/parents. The aim is to evaluate the effectiveness of the Therapeutic Conversation Intervention on caregivers of adolescents with ADHD. The result was, for example a significant improvement of quality of life.
Moen, Hedelin & Hall‐Lord, 2014. Norway, Sweden	Use of dialogue	Empirical study about the role of public health nurses (PHN) and families with a child with ADHD. The aim of the study was to explore the PHN role in relation to families with a child with ADHD. The paper points out the importance of building a good relationship with parents using dialogue and, continuity. Supervising parents also requires dialogue, and the PHN's support for parents and the entire family is important.
Bauer & Webster‐Stratton, 2006. USA	Importance of prevention by, for example parenting programmes	This paper reviews selected parenting programmes for children aged 2–8 years to inform the options available to families with children with behaviour problems. Parent training programmes are an effective option to promote positive parenting. It is essential to think not only of how to screen and treat, but also of how to prevent behavioural problems.

The results and knowledge from earlier studies (Barlow, Bergman, Kornor, Wei, & Bennett, 2016; Barlow, Smailagic, Huband, Roloff, & Bennett, 2012; Barlow & Stewart‐Brown, 2000; Bearss et al., 2015; Cavonius‐Rintahaka et al., 2019; Dretzke et al., 2009; Farmer & Reupert, 2013; Fosco, Sarver, Kofler, & Aduen, 2018; Kane, Wood, & Barlow, 2007; Peasgood et al., 2016; Trillingsgaard, Trillingsgaard, & Webster‐Stratton, 2014) have been taken into account during the development and implementation process of the DFG family intervention.

### 2.2 Theoretical basis for the DFG

The review of the literature revealed that while psychosocial interventions have been developed, the uniqueness and individuality of the specific family and its family members have received insufficient attention. The structure of rigid psychoeducational programmes does not necessary give space or time for family members to express their individual needs or to ask questions. Crises or other adversities may occur in all families; yet having a child with NDD can have different impacts on different family members. However, all of family members have an effect on each other and the family dynamic and communication inside the family are therefore crucial considerations when addressing family health (Cavonius‐Rintahaka et al., 2019).

Based on previous literature, studies and clinical experience, we believe that professionals need to find the right balance between psychoeducation and having a sensitivity to the voices of families and individuals speaking from experience. Dialogue with professionals and family members of a child with NDD only deepened our understanding of a family's vulnerability and their individual resources and needs. This knowledge has directly influenced the approach taken during the development of the DFG intervention. The medical and nursing knowledge behind DFG is a combination of understanding the complexity of NDD (Thapar et al., 2017) and ESSENCE (Gillberg, 2010) and then having the competence to transform it into practical guidance for families to help them in their daily life. Traditional background elements of family therapy such as *Open dialogue* (Seikkula & Trimble, 2005), *reflection* (Weingarten, 2016) and *systems therapy* (Haefner, 2014) are influencing DFG background theories. But especially, the dialogic approach is key to this family‐targeted intervention.

### 2.3 Development of the DFG family intervention

**FIGURE 1:**
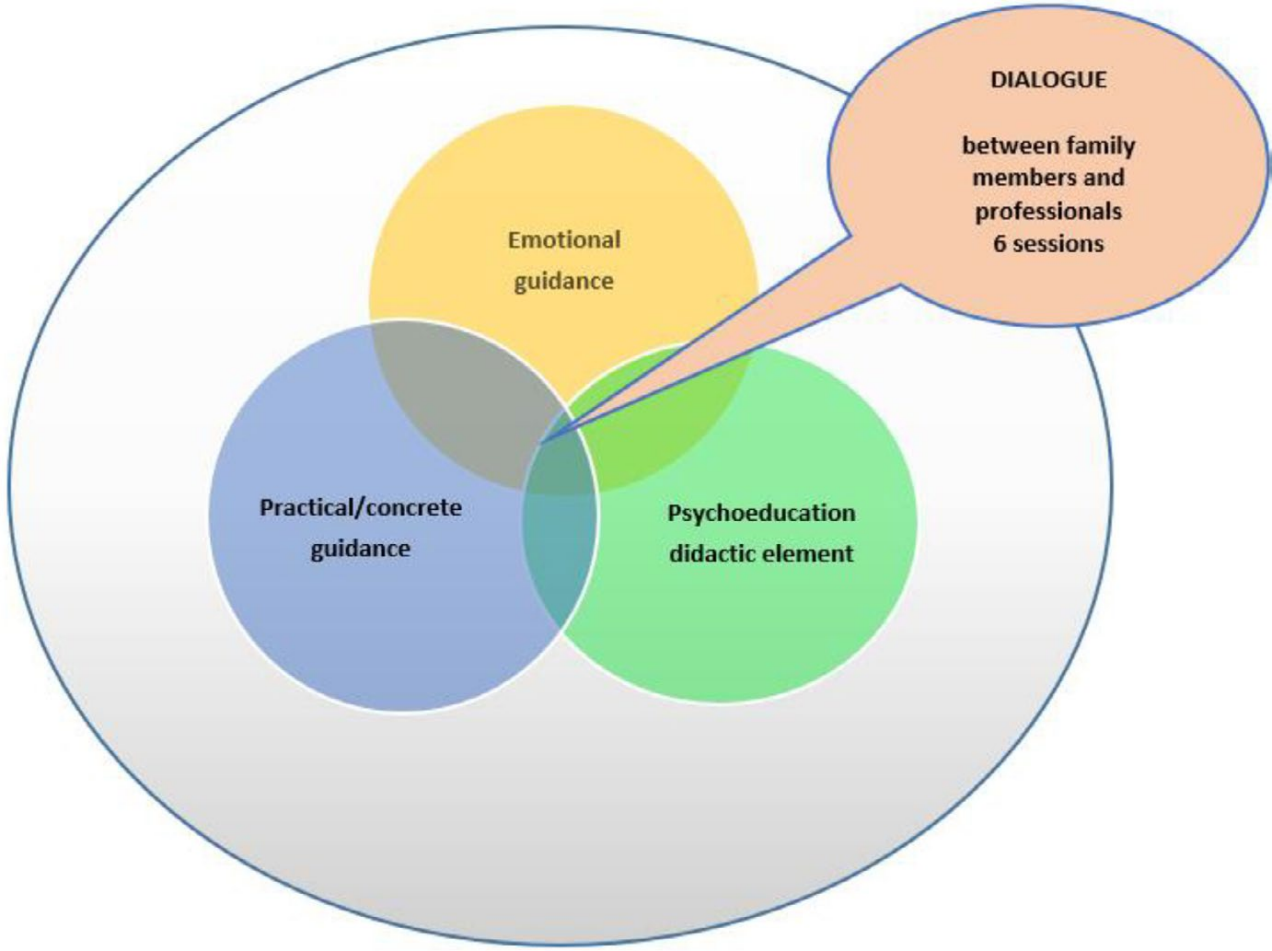
Content of the DFG areas

Dialogical Family Guidance is designed to help all family members to receive knowledge and gain an understanding of NDD/ESSENCE. DFG differs from other family interventions, because it targets on all family members and not just the parents. The DFG guidance areas (Figure 1) focus on both the need and benefit of psychoeducation, and giving continuous attention to the individuality of family members (practical and emotional). The DFG development process is seen as a combination of theory‐based research and knowledge based on clinical experience.

As mentioned earlier parents with children with NDD can have symptoms similar to their children due to the high degree of heritability (Thapar et al., 2017). Attention‐deficit/hyperactivity disorder symptoms in adults may present as inner restlessness, impatience and difficulties to sit still in meetings (Zalsman & Shilton, 2016). Poor time management skills can also appear, and these features need to be noticed, because the parent's own symptoms along with, for example impulsivity and attention disorders, can cause difficulties for parents to complete longer intervention processes. Low self‐concept might decrease parental expectations of being able to deal with emotional situations, and an experienced failure of emotion regulation might stabilize negative thoughts about oneself (Hirsch, Chavanon, Riechmann, & Christiansen, 2018). As mentioned before, psychoeducational interventions usually last 60–90 min including 4–6 sessions (Bauml et al., 2006). Accordingly, the DFG intervention includes six different sessions lasting 90 min per session. Given the issues mentioned above, any longer intervention process could potentially minimize the parents' own motivation and commitment, so establishing a time schedule for the sessions provides a sense of security for the family members.

### 2.4 Description of the DFG sessions and its three main components

The general approach in DFG is dialogical with an emphasis on collaboration between DFG therapists and family members to find solutions and make family resources visible. Using dialogue, DFG therapists gain knowledge about, for example the family system, parenthood, family crises and siblings´ reactions within the family. Open dialogue invites family members into a mutual learning process (Rober, 2010; Seikkula & Trimble, 2005). DFG offers a collaborative working process for all family members over six meetings within 3 months.

### 2.5 The three main components in DFG

Dialogical Family Guidance consists of three main components, (a) psychoeducational (didactic element) (b), practical guidance (skill training) and (c) emotional guidance including guidance and discussion about the personal, sensitive and unique experiences of family members. Dialogue between DFG therapists and families enables a response to families' unique needs regarding all three guidance areas (Figure 1). This also saves time when issues already familiar to the family do not need to be repeated.

**Psychoeducation** about NDD/ESSENCE. The goal of this component is to increase parents' knowledge about their child's special needs, diagnosis and symptoms, developmental factors and to increase their overall understanding of the child. Other family members' possible NDD symptoms are often of interest in this component because of the potentially strong hereditary impact. Worth noting is that this may be the first opportunity for parents to talk openly about these matters (Table 2).

**Table 2:** Content of the DFG family intervention areas

Practical and concrete guidance	Emotional guidance	Psychoeducation
Parents get concrete guidance, tips and advice for daily life with a child with special needs. All FM get concrete guidance for their daily life. Family gets help as a whole family unit. Dialogue about the child's special needs, special training, how achievements are seen at home, impacts on daily life. The family get guidance about child's neuropsychiatric/ESSENCE/NDD disorders and discussion of how it can impact all FM. Discussion with the family about their opinions about how the child's special needs specifically affect their family. FM get information and guidance on how they can participate in the habilitation of their child at home in daily life. Other surroundings (school, day care) and people (family network) are also included in the discussion.	Professionals collaborate with the family members (FM). Professionals listen to the FM and to their hopes/needs. Professionals show respect to the family because the family are willing to be active and participate in the habilitation of the child with special needs. Professionals show their concern for the well‐being of FM. Professionals are interested in the family as a unit. Dialogue about the child with special needs—personality, demands and resources. Resources of the family are mapped and made visible. Dialogue about parenthood, being parents together, being the child's mother and father. Professionals give enough space for family members to express their feelings. Professionals confirm parents' hope to have strength as parents and in parenthood. Professionals give positive feedback to the FM about participating and being concerned about the child's/siblings' daily habilitation.	Information about the child's symptoms, special needs, diagnostic procedures, diagnosis, treatment, therapy and habilitation. Repetition about the child's history regarding diagnosis and the current situation (medical history, appointments, hospitals, meetings with professionals, etc.). Guidance about the child's special needs, treatment, therapy and habilitation. Dialogue about the child's diagnosis, symptoms, rehabilitation, therapy, treatment, proceedings, achievements—the parent's (mother and father) perspective, knowledge, understanding are made visible. Information, education about the child's daily life needs. Professionals show interest in how the family is handling/managing daily life. FM get guidance about available assistance, support groups, habilitation facilities, outpatient clinics, social resources, etc. that are available. Parents get education about why structure and consistent guidance is important for the child's daily life. FM get guidance about neuropsychiatric/ESSENCE/ NDD disorders and commonly known impacts on family individuals and how family function can be affected. Information about important principles as a parent, parental roles and responsibility, parent‐child communication and interaction. Professionals give information to parents about the importance of their participation as family members in the everyday habilitation plan.

**Practical/concrete** guidance includes tailored guidance connected to the daily living of the entire family, to help parents to find solutions to their daily life and how they can meet the needs of a child with NDD while also meeting the needs of the rest of the family. In meetings where only the parents are present, attention is paid to both parents' individual desires, resources and habits. The goal of this component is to find common solutions suitable to both parents. Mothers and fathers operate and function from their own personal starting point, and therefore, the guidance is also personalized. A different approach is needed if there are children/siblings present (Table 2).

**Emotional guidance** includes DFG therapists being reflective and listening to family members' unique life situations without prejudice or pre‐held attitudes. One goal is to increase families' own activity and functionality, by making the family members' own resources visible. In this way, the family's overall well‐being can be increased. Emotional support is provided by listening and verbally supporting family members as they discuss their concerns and helping them to develop personal skills and abilities (Table 2).

#### 2.5.1 Family sessions 1–6

Session 1 is dedicated to practical arrangements termed as the *setting* (place, time schedule, planning and frequency of the meetings). This first session also includes dialogue about who is living in the family, the actual family situation and the NDD symptoms of the child. Only the parent(s) attend session 1, so as to allow them to talk freely about their actual family situation, their child's special needs, their concerns and what their needs and expectations are towards DFG. The DFG therapist will gain knowledge about what issues are important for the parents and what kinds of demands are the most acute to address during the DFG sessions. A preliminary plan is made during session 1, and the following sessions (2–6) follow themes from the DFG manual, taking into account the unique and individual needs of the family members. The themes covered in the manual are supposed to help the DFG therapist to bring up most common important issues concerning the focus group families. These themes are presented in Table 2.

After each session, the DFG therapist makes notes on the DFG checklist about which themes have been discussed and which themes still need attention. This assures that all three of the DFG guidance components and themes have been handled during the DFG sessions. This checklist policy provides a quality factor for the DFG family intervention and helps providers to take commonly important themes into discussion, while paying attention to the individuality of the family at the same time.

### 2.6 Training for professionals to become a DFG therapist

The DFG training course includes theory‐ and experience‐based knowledge. The topics of DFG training are presented in Table 3.

**Table 3:** DFG education programme components

Components	Core topics
Background & DFG implementation process	DFG development process
	Why is DFG needed?
	Educational goals
	Target group
	Administrative and inner setting
	DFG implementation process
Introduction to DFG education	Content
	Educational goals
	Time schedule
Parenthood	Parenthood—factors and skills needed
	Different roles as parent, spouse and person
Parenthood and a child with NDD (theory and praxis examples)	Feelings as a parent
	Stress
	Crisis
	Defence and coping mechanisms
NDD in the family (theory and praxis examples)	Family system theories
	When the parent has NDD
	Couples' relationship when the spouse has NDD
	Siblings' relationships
Introduction to family interventions (theory and praxis examples)	Common principles when working with couples and families
	Parent groups
	Incredible Years programme and literature presentation
	Family school
	Psychoeducation
	Psychotherapy/ family‐ and couple therapy
	Family evaluation
Introduction to dialogue (theory and praxis exercises)	Active listening
	Reflective attitude
	Use of family narratives
	Dialogue and dialogical attitude
	Social and emotional coaching towards dialogical working
DFG	Common principles in DFG
	Setting
	Goals when working with families
	Structure
	Manual
	Checklist
	Discussion about targeting DFG to the right families
Tips and materials that can be used with children and parents	Written materials are shared (can be used during the DFG process)

The educational goals for professionals are as follows:

1. That the principles and substance of DFG are well understood.
2. That participants increase their knowledge or confirm their own existing knowledge about family dynamics and parenthood in families with children with NDD, including emotional aspects.
3. That participants can proceed and perform DFG independently (or with another DFG therapist).

A pilot training course was carried out in 2014. Subsequently, five DFG training courses were held between 2014–2019 for a variety of professionals, mostly nurses. The total length of DFG training was 27 hr. The researcher conducted the DFG training courses and has acted as the clinical supervisor for DFG therapists. Participants received a certificate of training and can be called DFG therapists once their DFG training course was completed.

Dialogical Family Guidance training can also be seen as a way of updating education for professionals from various occupations (e.g. nurses, medical doctors, social workers, psychologists and psychotherapists). The expectation for DFG therapists is to follow the DFG principles and to avoid any modification when delivering DFG to the families. The aim is to keep the DFG content and structure genuine and to secure the original quality of the DFG programme.

### 2.7 DFG implementation

**FIGURE 2:**
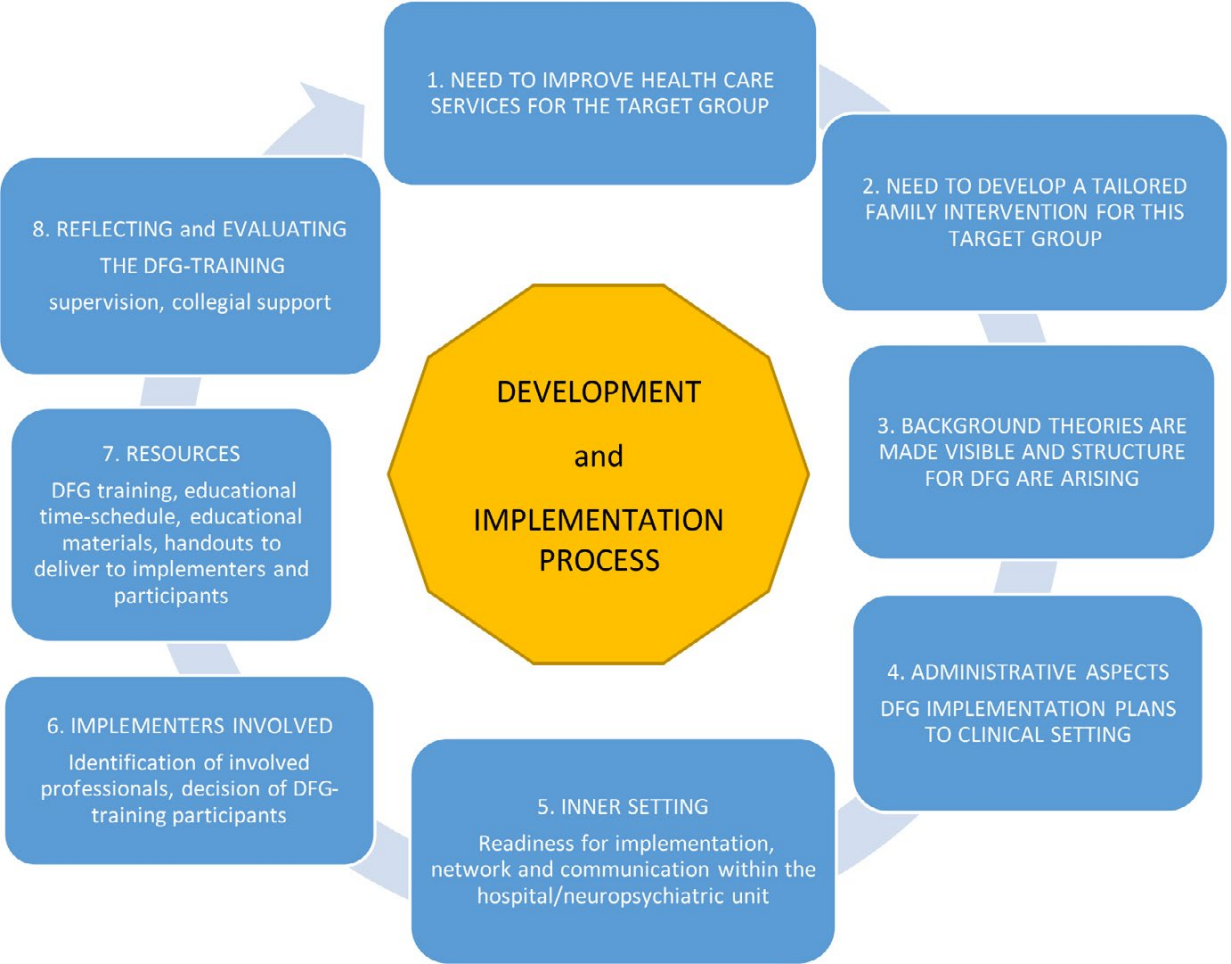
The DFG development and implementation process

Readiness for implementation to a clinical setting requires a lot of communication between actors on different administration levels. The personnel involved are in the best cases motivated to work alongside each other and cooperate during the implementation phase (Figure 2). The DFG initiative was launched in the children's neuropsychiatric unit of a university hospital in a clinic administered by child psychiatry. The DFG intervention implementation process started with administrative issues such as selecting suitable units, gaining permission from the administration, sharing information about this new family intervention model and initializing the recruitment of suitable professionals to the DFG education programme. The realization of DFG courses and how the clinical supervision of professionals would be administered was included in the implementation process. This was important as nurses and other professionals proceeded with their training and prepared for independent work as DFG therapists. In some case (mainly involving nurses), the professional's job description needed modification to assure their possibility to proceed with the DFG intervention after DFG training as a part of their clinical work. The professionals own motivation and willingness to attend DFG training were seen as an important selection criteria.

Approval from the hospital ethical board was applied for, so as to be able to test the effectiveness of DFG in the future from the families' point of view. Acceptance from the hospital ethical committee of psychiatry (106/13/03/03/2012) and research approval from the hospital board was received from 2012–2019.

A manual has been created to help DFG therapists in their work. The manual includes six different themes which can be used flexibly during the DFG sessions. The manual gives structure to the DFG process, and using the manual has been felt to be highly beneficial by DFG therapists.

Data regarding the experiences and satisfaction of DFG training were collected with the consent of those who participated. A total of 44 professionals completed the DFG training (2014–2019) and 26 of those (59%) answered a questionnaire mostly consisting of questions about their opinions about the DFG training they had received. Together with two background data questions, eight questions asked about how the training had aided their work with families, given them tools to use in practice, a new understanding about focus group families, new understanding about the importance of dialogue and whether they would you recommend DFG for families and DFG training to other professionals. The survey data were analysed using the SPSS statistical programme.

## 3 RESULTS

At this hospital, nurses and social workers completed the education programme between 2016–2019. However, it is mainly nurses, often working in pairs with families during the DFG process. This helps professionals to learn and internalize this new intervention and minimize their own tension. Participating on the DFG training course seemed to increase the possibilities of a more independent role as a nurse being able to deliver the DFG intervention to families.

The education programme offered the possibility to rehearse the dialogue that can be used in practice. The use of dialogue was also an important pedagogical method giving experiences of being listened to during the DFG training process. Comments, questions and the narratives of participants were important dialogical elements that featured in the DFG training. Worth noticing in this project was that using dialogue can be difficult for some professionals. Also, the expectation of taking all of the family members into consideration, instead of focusing only on the child with NDD, can be demanding. Therefore, one assumption is that not only experience and skills, but also the personality of the professionals involved, affects how DFG is delivered for the family.

The analysis of the questionnaire completed after DFG training reported a high level of satisfaction concerning the training itself and the question “*Would you recommend the DFG education to other professionals”* was answered “absolutely yes” or “yes” by 96% of respondents. Regarding their perceptions of the usefulness of DFG initiative itself, the question “*Would you recommend DFG for families”* was answered “absolutely yes” or “yes” by 100% of respondents.

The analysis of the open‐ended question (“*Can you tell about your experiences and suggestions to improve the DFG training programme”*) revealed that the professionals' personal experience of being heard during their DFG training increased their understanding of how parents can feel when DFG therapists are listening to them. The participants' experience was that the theory‐based parts of the education became more understandable when they connected to the participant's own narratives during their DFG training. DFG training gave them a new approach to delivering knowledge and understanding to families using dialogue, including tailored psychoeducation and emotional and practical guidance. The DFG training programme was felt to be professionally delivered, comprehensive and well structured. DFG training participants also appreciated having an opportunity to share their experiences with other professionals.

## 4 DISCUSSION

This article describes the development and implementation of a Dialogical Family Guidance intervention, aimed at families with a child with NDD. As previously mentioned, the DFG development and implementation process evolved from clinical experiences involving parents' narratives, and drawing from data from a pilot study (Cavonius‐Rintahaka et al., 2019). Forming a functioning family intervention for this target group and implementing it successfully in clinical practice has been a long‐term project.

Reflecting previous knowledge and literature, there is no doubt that family interventions are needed for families with a child with NDD, especially when a specific demanding behaviour is involved (Dykens, 2015; Post et al., 2017). It is also well known that information about symptoms and diagnoses, as well as tips and advice when operating with these children in daily life are important parts of psychoeducation initiatives (Bauml et al., 2006; Nussey et al., 2013). But this knowledge alone seemed not to be enough. According to families on whom the demands of not only taking care of the child with special needs, but also the siblings and the relationship between parents had an impact, raising children with NDD is challenging for parenthood and over time there are risks to the parents' own mental health in terms of anxiety and depression if they do not receive help (Falk et al., 2014). Thus, there seemed to be a need to develop family‐focused approach including dialogue with all of the family members. The DFG intervention development looked to improve the quality of life of all family members.

The development of DFG (and especially the implementation process) has improved the attitude of nurses and other professionals to realize the wider impact the child's NDD has on the entire family. Listening to the voices of families plays an important part in understanding families of children with NDD or other disabilities (Post et al., 2017). Therefore, DFG therapists can be seen to be in a unique position to promote the health of all family members. This insight was strengthened among professionals during the DFG development and implementation process, and it seems that the implementation of DFG provided greater understanding for the whole team to make common efforts towards providing more family‐centred care at the unit.

Gathering the knowledge from earlier studies and existing interventions for this target group and combining it with clinical experience was a long, but necessary starting point. Before identifying the DFG components, a multitude of decisions were systematically made and a pilot study was carried out 2012–2014 (Cavonius‐Rintahaka et al., 2019). The intervention development process described here included many phases, and each activity made DFG development and implementation more meaningful. The ultimate goal was to develop an intervention that is theoretically based, acceptable to the target group and suitable as a systematic family intervention that could be used continuously at the clinic. In light of the experiences gained during the development and implementation process, we have succeeded with this goal. During the implementation process, it appeared that DFG is a feasible intervention for delivery in the community and in different healthcare settings. Additionally, DFG can also be viewed as a treatment that can be used across a range of paediatric diagnostic labels, because there are many common and similar background factors and stressors that are similar in families of children with different kinds of special needs.

The next step in this project is to conduct a study that will clarify the effectiveness of DFG in families with a child with NDD. There is also a further need to study whether DFG has positive implications for children with NDD and whether the family function in daily life, family dynamics and family health improves as a result of the intervention. To gain accurate knowledge of the impact of DFG has on families requires randomized, controlled studies to gain knowledge of DFG's effectiveness and efficacy and such a study is in progress and will be analysed using validated tools. In detailing the development and implementation of the DFG intervention, this paper can hopefully provide guidance for nurses and other professionals aiming to develop new mental health interventions for families with a child with NDD.

### 4.1 Limitations

Relatively, few professionals have taken part in the DFG training course, although many more are on a waiting list for DFG training. Unfortunately, the DFG training course evaluation received responses only from 26 of the 44 professionals who had taken part, so the lack of depth in data makes it too early to know whether the training course needs modifying, although preliminary experiences and feedback from participants were very good. The DFG implementation process described in this paper relates only to one university hospital, and therefore, the results cannot be generalized. A more long‐term perspective concerning DFG training experiences and expanding the implementation process to several units could give a wider perspective. Also, the experiences of DFG therapists of delivering the DFG in clinical practice to families would give important additional knowledge about the individual components of the intervention, and its overall impact. More objective knowledge about how the child with NDD and the rest of the family are affected by the DFG intervention would have given this paper more impact; however, this study is in progress and once complete; findings will be used to modify the DFG if necessary.

## 5 CONCLUSIONS

The published literature and clinical experience indicated a lack of a family‐focused intervention including dialogue for this particular target group. The development and implementation of the DFG initiative answered this need. The DFG training has received a positive reception among professionals who have taken part, and this has helped the implementation process in the clinical setting. Also administrative actors on different levels at the university hospital positively facilitated the implementation of the initiative. Overall, the DFG development and implementation process have improved the attitude of nurses and other professionals towards realizing the wide impact of the child's NDD on the entire family.

### 5.1 Relevance to clinical practice

This paper presents the DFG family intervention development and implementation processes, together with the details of the DFG education process and programme components. This information can be useful to nurses working with similar families and clinical surroundings, but the information can be applied by various professionals working in a setting that involve families with a child with NDD. This paper can offer tips to developers working in different areas to help them develop their own family interventions and implement them in different units. This paper hopefully increases the awareness of the importance of offering these families dialogical interventions that include all family members.

## CONFLICT OF INTEREST

No conflict of interest has been declared by the authors.

## AUTHOR CONTRIBUTIONS

DC‐R, ALA, EB and CG: Study design. DC‐R and ALA: Data collection. DC‐R, ALA and EB: Data analysis. DC‐R, ALA, EB and CG: Manuscript writing. All authors listed (a) meet the authorship criteria according to the latest guidelines of the International Committee of Medical Journal Editors. (b) all authors are in agreement with the manuscript.

## Supporting information

App S1

## Data Availability

Data are available on request from the corresponding author.
