# Supplementary material for: Dialogical Family Guidance (dfg)—Development and implementation of an intervention for families with a child with neurodevelopmental disorders
Source: Nurs Open. 2020 Sep 17;8(1):17–28. doi: 10.1002/nop2.627 (PMC7729547; doi:10.1002/nop2.627)
Supplement: Supplementary file 1 — App S1 [file NOP2-8-17-s001.docx]

**Revised Standards for Quality Improvement Reporting Excellence (SQUARE)**

1. **Title:**

“Dialogical Family Guidance (DFG) – Development and Implementation for Families with a Child with Neurodevelopmental Disorders.”

This manuscript is aimed to improve healthcare for families with children who need special care. Using Dialogical Family Guidance (DFG) with families helps professionals to focus on important issues (based on literature / quality), but also to be in dialogue with all family members to understand the uniqueness of all families (patient-centeredness). In this way all families do get the information and help they need.

1. **Abstract:**

a. *Background*; Studies of interventions targeted to families with children with neurodevelopmental disorders (NDD) highlight the need to focus on family health, dynamics and family functionality, along with psychoeducation. Dialogue between professionals and families should be given attention. Development and implementation of a family intervention called Dialogical family guidance (DFG), aimed for this target group is presented.

b. *Local problem*; There is no systematic way to help families with a child with NDD in practice and the result is confused families who experience that they are not getting any help at all.

c. *Methods*; The development of the different phases of DFG (including a literature review) and theoretical issues concerning DFG in clinical practice are outlined. Furthermore, the clinical experiences of DFG implementation are presented.

d. Interventions; this new intervention is DFG developed for families with a child with NDD. The manuscript describe the development process of DFG, as well as the implementation process.

e. *Results*; Development and implementation descriptions can be useful for service providers working in similar clinical surroundings. The intervention described can be applied in the clinical setting by various occupations where target group families are met. Overall, this article can be seen as offering a supplemental initiative to support traditional clinical work.

f. *Conclusion*; This information can be useful to providers working with similar families and clinical surroundings. The information shared in this paper can be applied by professionals working in a clinical setting with families with a child with NDD, and gives tips to develop their own family interventions and implementation processes. This paper further serves to increase awareness of the importance of offering these target families interventions that consider entire families, and also the importance of using dialogue when implementing them.

1. **Problem description**

Families with a child with NDD experience that they do not get any help, or sufficient help or the answers to their questions from health care service.

1. **Available knowledge**

There are studies and literature where interventions have been used to get more knowledge about what kind of knowledge families in this focus group needs. Psychoeducation is well studied and there is consent about psychoeducation as an important help for these families. However there are not so much knowledge about *how* professionals should give this information. There are studies giving lists and systematic tables about the content of information, but studies are needed about the fact that all families do not need the same kind of information because they have different needs and expectations.

1. **Rationale**

There are family interventions to this target group (eg.Incredible Years, The Triple P), but as families gave a lot of feedback to professionals that eg. the siblings were not notified and that the interventions gave a lot of information, but did not give tools how to manage difficult situations at home. This information from parents gave professionals a need to develop other kind of family intervention.

1. **Specific aims**

The aim of this report is to deliver knowledge to other professionals about how DFG was developed and how DFG has been implemented to hospital surrounding. This report gives us preliminary knowledge also about how this new family intervention DFG is working in clinical surrounding.

1. **Context**

The context is families with a child with NDD, neuropsychiatric disorders. In practice, many children have both neurological and psychiatric disorders. The context in this report is an neuropsychiatric unit at an University hospital. This unit is administrative under child psychiatry.

1. **Intervention**

This paper present the intervention DFG in detail. Also the educational part of professionals getting DFG education and what the education is including is presented. The implementation process is bringing up issues about administration and identification of involved professionals. The total information in this report makes it possible for others to reproduce it in their surroundings.

1. **Study of interventions**

The paper includes a literature review to present a wider perspective to this specific issue about family interventions and families with a child with NDD. Common principals and used methods are presented and discussed.

1. **Measures**

This paper is presenting the development and implementation process and the measurement about families experiences are not measured yet. However there are a measurement about professionals` experiences about the DFG education. The next paper is including measurement about how DFG is helping families and children from their point of view. The data is already been collected.

1. **Analysis**

The outcome of professionals experiences were collected by using a questionnaire. The answers were put in SPSS program and analyzed in that way.

1. **Ethical considerations**

The leaders of the hospital were from the beginning motivated and helping in many ways to get DFG education to hospital staff (mainly nurses, but also therapists, doctors and social workers). Also the following implementation process was a natural decision from all involved administrative and clinical staff.

DFG education, implementation and using DFG with families got acceptance from the hospital ethical board (number: 106/13/03/2012, updated 26.3.2015).

1. **Results**

The result of DFG implementation is presented in this paper. There are many administrative issues to discuss and the experiences of professionals taken part of DFG education- and implementation process is mainly positive. Also, families taking part have expressed their gratitude and satisfaction. Families experiences mentioned in this paper are although only preliminary and not official measured yet. As mentioned before, a controlled intervention study is already in process and the results of families` and children`s experiences of DFG is going to be in published later.

1. **Summary**

Because the development and implementation is already been in process for a few years in this particular unit, the strength of DFG has been noticed and claimed as an ordinary family intervention at this unit. Key findings are the satisfaction of leaders and clinical professionals including different occupations. The experience is that DFG is suitable for clinical settings on different levels in the community. Professionals have been satisfied with the DFG education and feel they are more ready to help the focus group families.

1. **Interpretation**

Other publications support the experiences that have been visible during development and implementation processes. Also the findings from other studies regarding families` experiences about family interventions have been noticed during the development of DFG.

1. **Limitations**

Because DFG has not yet been studied as a controlled study yet, it is not possible to know so much about the effectivity from parents` and children`s point of view. Also the amount of professionals getting the DFG education and using it in their work, is small. Although there are a lot of interest towards DFG, we need more experiences from personnel and a follow-up studies for families.

1. **Conclusions**

It is clear already by now, that the hospitals and private therapists and social care have been taking DFG to their working procedures. This tells us that DFG has been experienced as an functional tool to professionals working with families with children with NDD.

1. **Funding**

There has not been any funding for the development and implementation process of DFG, but the hospital leaders have been accepting all the work to be done during working hours. This has made the educational and implementation processes easier to fulfill.
